# Ultrasound of the lateral femoral cutaneous nerve in asymptomatic adults

**DOI:** 10.1186/1471-2474-13-227

**Published:** 2012-11-22

**Authors:** Jiaan Zhu, Yiwen Zhao, Fang liu, Yunxia Huang, Junjie Shao, Bing Hu

**Affiliations:** 1 Department of Ultrasound, Shanghai Jiaotong University Affiliated Sixth People's Hospital, Shanghai Institute of Ultrasound in Medicine, 600 Yishan Rd, Shanghai, 200233, China; 2 Department of Ultrasound, Central Hospital of Fengxian District, 9588 Nan Feng Road, Shanghai, 201400, China; 3 Department of orthopedics, Shanghai Jiaotong University Affiliated Sixth People's Hospital, 600 Yishan Rd, Shanghai, 200233, China; 4 600 Yishan Rd, Shanghai, 200233, China

**Keywords:** Ultrasound, Lateral femoral cutaneous nerve, Inguinal ligament, Anatomy

## Abstract

**Background:**

To define the sites where the lateral femoral cutaneous nerve (LFCN) is more easily visualized and to describe the anatomical variations of the LFCN.

**Methods:**

A total of 240 LFCNs in 120 volunteers were evaluated with 18 MHz ultrasound; the intermuscular space between the tensor fasciae latae muscle and the sartorius was used as an initial sonographic landmark. The time taken to identify the nerve was recorded. The number of nerve branches at the level of the inguinal ligament (IL) and the relationship between the LFCN and the IL was assessed. The nerve cross-sectional area (CSA) of the LFCN and the distance between the LFCN and the anterior superior iliac spine was measured.

**Results:**

Each nerve was identified using ultrasound in all participants. The mean time for identifying the nerve was 7s for unilateral LFCNs. The nerve passed under the IL in 198 cases, whereas in 44 cases, it passed through to the IL. The LFCN consisted of 1–4 branches just after its passage under or through the IL. The CSA of the LFCN was 1.04±0.44 mm^2^, and the mean distance between the LFCN and the anterior superior iliac spine was 15.6 ± 4.2 mm.

**Conclusions:**

It is easier to identify the LFCN if the intermuscular space between the tensor fasciae latae muscle and the sartorius is used as an initial sonographic landmark. The anatomical variation of the LFCN can be viewed with high-frequency ultrasound.

## Background

The lateral femoral cutaneous nerve (LFCN) is a pure sensory nerve that arises from the L2 and L3 spinal nerve roots and travels downward lateral to the psoas muscle, and then crosses the iliacus muscle. Near the anterior superior iliac spine (ASIS), the LFCN then passes under or through the lateral aspect of the inguinal ligament (IL) and over the sartorius muscle into the thigh. Running caudally, the LFCN enters a lenticular compartment between the sartorius muscle and the tensor fasciae lata muscle, formed by a double layer of the fascia lata [1]. The LFCN has received much attention because of its association with meralgia paresthetica [1,2]. Knowledge of its anatomical variations is also crucial for preventing nerve injury during the insertion of needles into the ASIS [3], the harvesting of the anterior iliac crest bone grafts [4], and other surgical procedures [5]. LFCN grafts can also be used to repair facial nerve injuries and soft-tissue defects [6,7].

Because of the anatomical variations in the course of the LFCN, it may be difficult to identify and protect it during surgical dissection [4,5,8]. The ASIS is a classical landmark for the LFCN in some surgical procedures and in regional anesthesia that involve nerve blocks for the treatment of meralgia paresthetica. However, the rate of successful anesthesia has only been approximately 40% based on the use of anatomical landmarks [9].

It is useful to visualize the LFCN for clinical practice. There are several promising studies about the ability of ultrasound in the evaluation of the LFCN [10-12], the use of ultrasound guidance in regional anesthesia for blocking the LFCN [13-16] and the use of ultrasound in nerve conduction studies of the LFCN [17]. These ultrasonographic techniques involved the use of the ASIS, the IL, and the sartorius muscle as the landmarks to identify the LFCN. However, because of its small size and the similarities in the echo characteristics between the LFCN and the IL with 10–14 MHz ultrasound, it is not always easy to distinguish the LFCN from the surrounding tissues using ultrasound. In addition, to the best of our knowledge, ultrasound studies on the anatomical variations and the reference values of the LFCN are lacking in the literature. The main objectives of this study are to define the sites where the LFCN is more easily visualized and to describe the anatomical variations of the LFCN.

## Methods

This study met the guidelines set by the ethics committee of the Shanghai Sixth People’s Hospital (Shanghai, China). All subjects were selected randomly and gave informed written consent for their inclusion in this study.

### Subjects

A total of 120 subjects were prospectively included in the study. All study subjects were asymptomatic volunteers without any symptoms of pre-existing pain or dysesthesia in the distribution of the LFCN. Subjects with diabetes mellitus, pregnancy, peripheral neuritis, and a history of injury or surgery on the spinal column, pelvis, or the groin were excluded from the study.

### Ultrasound

Each subject was positioned supine and was scanned bilaterally by an investigator (J.A., 10 years of neuromuscular ultrasound experience) using the Mylab 90 (Esaote, Genoa, Italy) with an 18 MHz linear array transducer.

**Figure 1:**
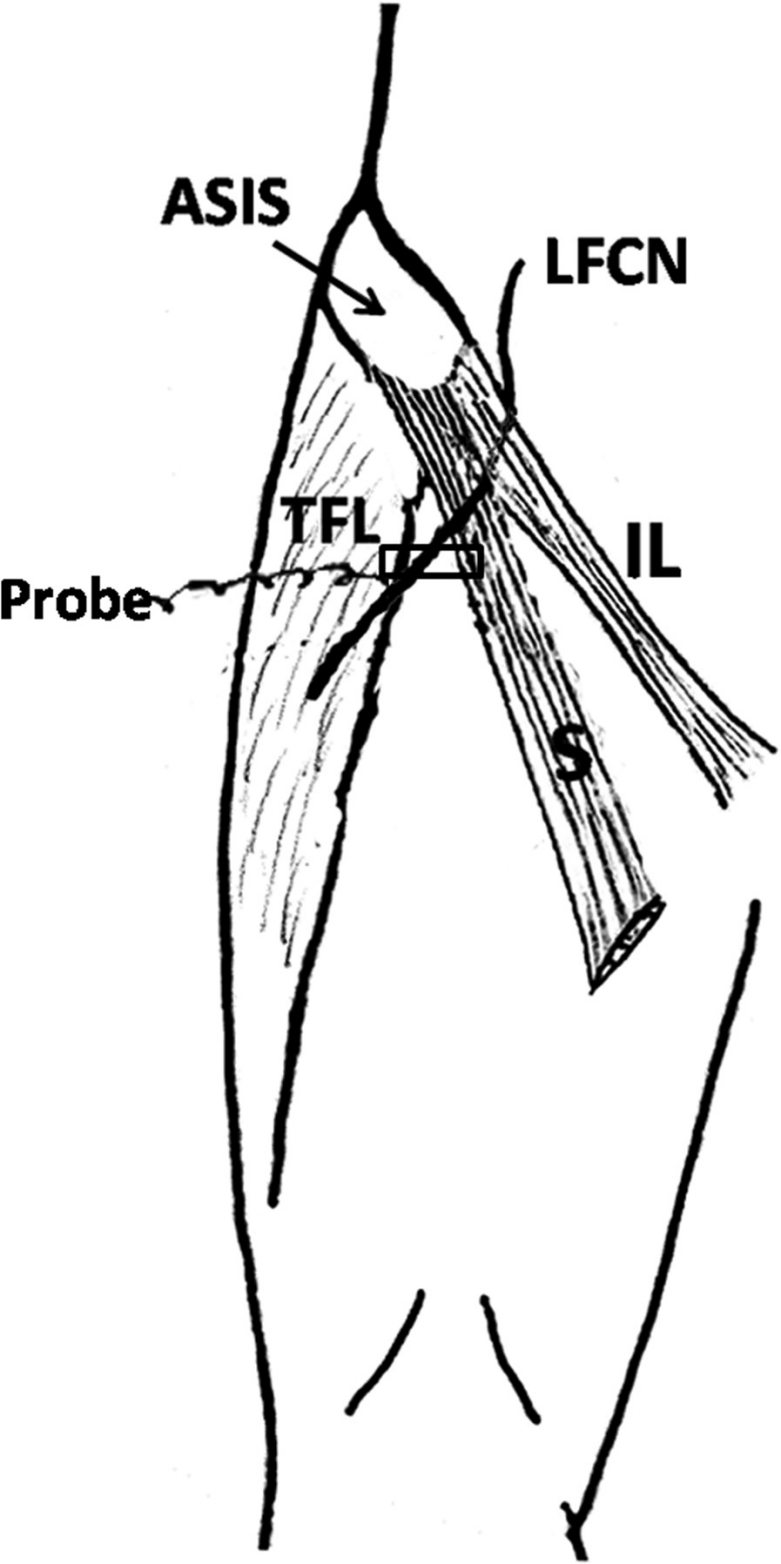
**Schematic diagram showing the course of the LFCN and the initial location of the probe.** ASIS: anterior superior iliac spine; LFCN: lateral femoral cutaneous nerve; TEL: tensor fasciae latae muscle; IL: inguinal ligament; S: Sartorius.

**Figure 2:**
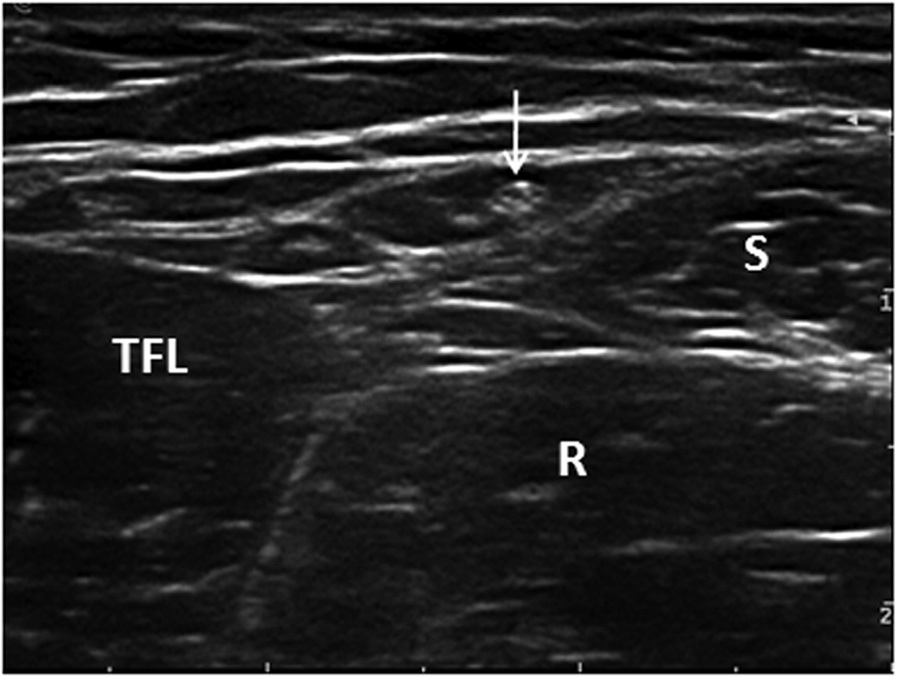
**Transverse ultrasound image of the LFCN lying within the intermuscular space between the tensor fasciae latae muscle and the sartorius.** LFCN: lateral femoral cutaneous nerve; TEL: tensor fasciae latae muscle; S: Sartorius; R: rectus femoris.

The ultrasound transducer was placed in the transverse position and was first placed 1–2 cm distal to the lateral IL. Initially, the tensor fasciae latae muscle and the sartorius were imaged (Figure 1). To verify the tensor fasciae latae muscle, the transducer was sometimes moved distally. As the distal tensor fasciae latae muscle inserts into the iliotibial band, the echo of the muscle of the tensor fasciae latae muscle should sonographically disappear. The LFCN was then identified in the intermuscular space between the tensor fasciae latae muscle and the sartorius. Because there was substantial contrast between the echo characteristics of the LFCN and that of the surrounding tissue, the LFCN could be easily identified and usually showed an ovoid hypoechoic structure with hyperechoic dots within it (Figure 2). which was similar to the other of the peripheral nerve. The nerve cross-sectional area (CSA) at this site was measured bilaterally in all 120 participants. When the LFCN was identified using ultrasound and then the angle of incidence of the probe was adjusted until it was perpendicular to the nerve, the smallest cross-sectional image was obtained. Because the CSA of the LFCN is so small that the trace function on the ultrasound device is unable to trace the nerve, the CSA was calculated from an area formula for ovals (CSA= Pi * A * B *1/4, Pi=3.14, A=the longest side of the oval, B=the shortest side of the oval). If the LFCN had several branches, the CSA of the anterior branch was measured. The CSA was measured three times, and the mean value was recorded. Then, the transducer was slowly moved proximally along the LFCN to the IL. The IL is noted as a linear hyperechoic structure running from the pubic tubercle to the ASIS [12,18]. The number of nerve branches at the level of the IL and the relationship between the LFCN and IL were assessed. The distance between the LFCN and the ASIS was also measured. By observing the course of the nerve, the nervous structure assed was identified LFCN and not another nerve structure.

To test the difficulty or ease of using our protocol to detect the LFCN, the other two radiologists (Y.X. and F., who both had 2 years of neuromuscular ultrasound experience) were trained by J.A to perform the ultrasound examination procedure for inspecting the LFCN, and the time taken to identify the nerve was recorded. Intraobserver reliability was evaluated in 30 cases that were selected by the radiologist (Y.X.).

### Statistical analyses

All parameters were compared bilaterally. Numerical and categorical variables were compared by independent unpaired t-tests and by the Chi-squared test, respectively. A regression analysis of the data that compared age, sex, height, weight, and the distance between the ASIS and LFCN was performed. The intraobserver reliability was assessed according to the kappa coefficient. A p value of less than 0.05 was considered significant.

## Results

All 120 subjects were Chinese (72 (60%) were male) with a mean age of 41.4 ± 21.3 years (range 18–76), a mean height of 173 cm, a mean weight of 71.1 kg, and a mean BMI of 24.9 ±4.1.

Each nerve was identified using ultrasound in all participants. In the intermuscular space between the tensor fasciae latae muscle and the sartorius, which presented a hypoechoic structure, the transverse sonogram of the LFCN usually showed an ovoid hypoechoic structure with hyperechoic dots within it (Figure 2).

**Figure 3:**
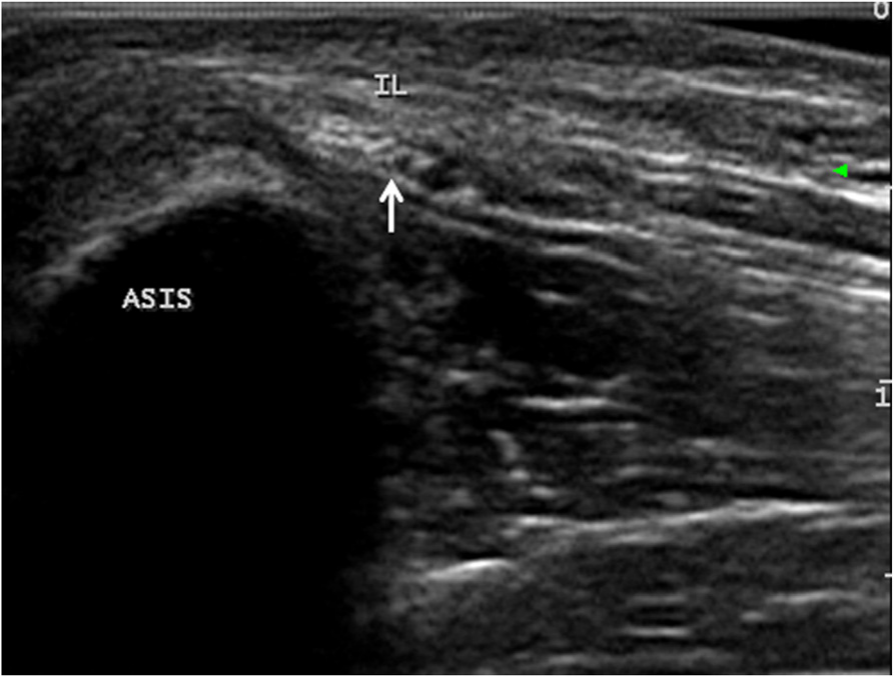
**Transverse ultrasound image shows the nerve passing under the inguinal ligament.** ASIS: anterior superior iliac spine; LFCN: lateral femoral cutaneous nerve; IL: inguinal ligament.

**Figure 4:**
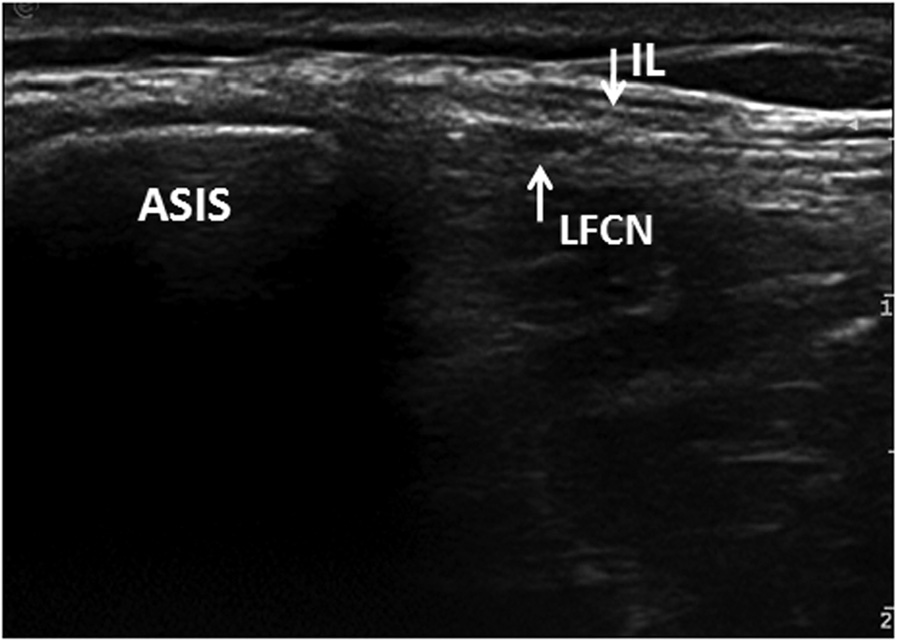
**Transverse ultrasound image shows the nerve passing through the inguinal ligament.** ASIS: anterior superior iliac spine; LFCN: lateral femoral cutaneous nerve; IL: inguinal ligament.

No significant differences were observed between the right and the left sides with respect to the CSA, the distance between LFCN and the ASIS, the numerical and categorical variables of the LFCN, or the relationship between the nerve and the IL. Therefore, the parameters from the bilateral sides were combined for the final analysis. The CSA of the LFCN was 1.04±0.44 mm^2^. The mean distance between the LFCN and the ASIS was 15.6 ± 4.2 mm (range 2.2–38.7). The nerve passed under the IL in 198 cases (82.5%), whereas in 44 cases (17.5%), it passed through the IL (Figures 3, 4). The LFCN consisted of 1–4 branches just after its passage under or through the IL. The nerve was represented by a single structure in 174 cases (72.5%) and consisted of 2, 3 and 4 branches in 36 (15%), 27 (11.3%) and 3 cases (1.2%), respectively. No statistically significant differences were observed between the ultrasound findings and age, sex, height, weight, or BMI.

**Figure 5:**
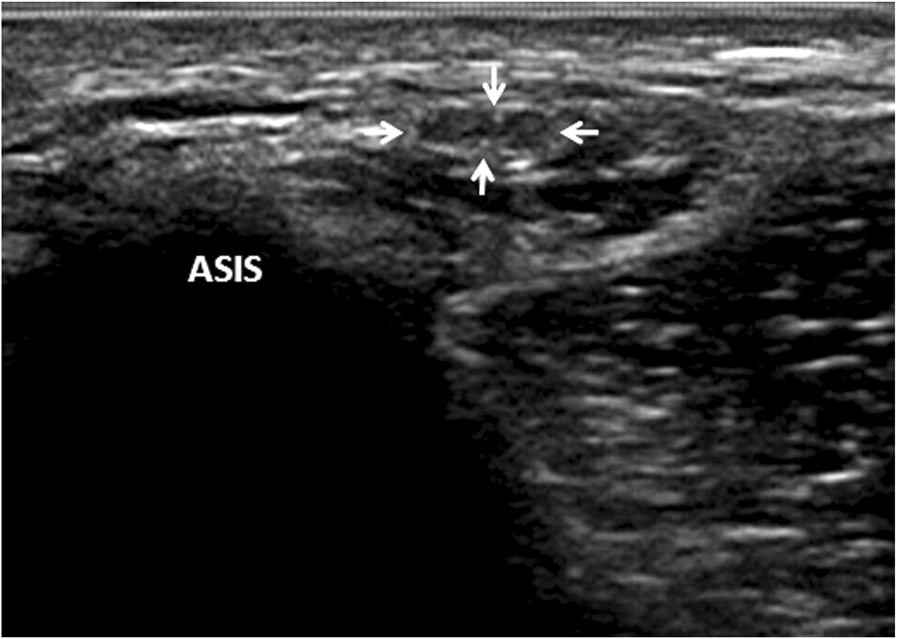
**Transverse ultrasound image shows a neuroma (arrow) of the LFCN.** ASIS: anterior superior iliac spine.

Two neuromas were identified in 2 volunteers, all which were located close to the ASIS (Figure 5).

The mean time required for identifying the nerve (the duration from placement of the ultrasound transducer on the skin to visual identification of the unilateral LFCN) was 7s. In the same 30 cases, the mean time was 9s (F.) and 10s (Y.X.). The intraobserver variability correlation coefficient was 0.912.

## Discussion

Our study suggested an easier method for identifying the LFCN in volunteers using the ultrasound technique. There have been several reports about the ultrasonographic appearance of the LFCN [10-17]. Irene and colleagues [10] used the two fascial layers as the initial sonographic landmark to locate the LFCN and the mean time of identifying the LFCN was 22.5s. Bodner and colleagues [11] identified the LFCN based on the landmarks of the IL and ASIS in a cadaver and in 8 volunteers, but they did not visualize all the LFCN. Tagliafico and colleagues [15] described the technique to identify the LFCN based on the ASIS landmark in 20 patients; however, the average time for the procedure was 12 minutes. The same method was used for imaging the LFCN by Hara and colleagues [16] for ultrasound-guided LFCN blocks. Some researchers [12-14,17] reported that the most effective technique for identifying the LFCN was to search for the site at which it crossed the sartorius muscle. However, the LFCN is a very small structure that is somewhat difficult to differentiate from the surrounding soft tissues at this site, so the nerve was not observed in 30% of the cases in Damarey’s report [12]. In ultrasound examinations, anatomical structures are more easily differentiated if the different tissues have greater echo differentiation on the sonogram, which is especially important for identifying tiny structures. The intermuscular space between the tensor fasciae latae muscle and the sartorius presents a hypoechoic structure, which has an echo signature quite difference from the nerve. Therefore, we recommend that the intermuscular space between the tensor fasciae latae muscle and the sartorius can be used as an initial sonographic landmark to scan the LFCN. Another reason for easier imaging of the IFCN in this study might be the use of an 18 MHz ultrasound transducer, which has a higher resolution. Based on our study results, just 7s was required to successfully identify the LFCN in all subjects, which was obviously shorter than the 22.5 seconds [10] or the 12 minutes [15] required in previous reports.

Once the LFCN was identified, the course of the nerve was then traced by scanning the structure proximally and distally. Therefore, the relationship between the LFCN and the IL was clearly observed. As demonstrated by our results, a great variability was observed not only for the nerve number but also for the relationship between the nerve and the IL. In our series, the majority of LFCNs passed under the IL and included one or two branches passing at the level of the IL. This variability is consistent with previous anatomical studies [19,20]. These findings may have potential relevance to everyday clinical surgical practice. Knowledge of the relevant anatomy is crucial before planning a surgical approach to the LFCN and in regional anesthesia when blocking the LFCN for the treatment of meralgia paresthetica [3,4,13-16]. To the best of our knowledge, there has not been a previously published study of the relationship between the LFCN and the IL using ultrasound. The morphological information will facilitate the success of procedures that involve this nerve. In this study, the distance between the LFCN and the ASIS was measured. Kosiyatrakul et al. [4] reported the distance between the LFCN and the ASIS with 7 ± 7 mm (range 1-32 mm) which was relatively shorter than our data with 15.6 ± 4.2 mm (range 2.2 - 38.7 mm). Damarey B et al. [12] reported this distance with 24 mm (range, 3–50 mm). The different measurement results might be resulted from the anatomical variation of the LFCN. Further, we also measured the CSA of the LFCN, which may be helpful in the evaluation of meralgia paresthetica.

Neuromas of the LFCN have been paid attention in previous reports [12,21-23], and the neuromas may result from the mechanical microtraumas [21], chronic irritation, or the role of ilio pubic tract and deep circumflex iliac artery in nervous compression [23]. However, the detection rate of neuromas in this study was lower than previously reported. This difference may be result from research subject differences.

The main limitation of this study is that verification data were not available. Another potential limitation of the study was the possibility of inaccurate nerve number counts due to the smaller diameter of the LFCN. Further studies are needed to clarify these limitations. In this study, the use of an 18 MHz ultrasound transducer allows a clear image of the LFCN, but the disadvantage is that 18 MHz ultrasound transducers are not widely used. However, we assume that this method of scanning the LFCN could also be applied using low-frequency ultrasound.

## Conclusions

It is easier to identify the LFCN if the intermuscular space between the tensor fasciae latae muscle and the sartorius is used as the initial sonographic landmark. The anatomical variations of the LFCN can be viewed with high-frequency ultrasound.

## Competing interest

The authors declare that they have no competing interest.

## Authors’ contributions

JAZ, YWZ, and FL were involved in the design of the study and performed the statistical analysis. JAZ, YXH, JJS and BH were responsible for drafting the paper and revising it. All authors commented on the draft. All authors have read and approved the final manuscript.

## Pre-publication history

The pre-publication history for this paper can be accessed here:

http://www.biomedcentral.com/1471-2474/13/227/prepub
